# Mobile Health App for Tuberculosis Screening and Compliance to Undergo Chest X-ray Examination Among Presumptive Cases Detected by the App in Myanmar: Usability Study

**DOI:** 10.2196/37779

**Published:** 2022-06-07

**Authors:** Kyaw Ko Ko Htet, Aye Nyein Phyu, Thandar Thwin, Virasakdi Chongsuvivatwong

**Affiliations:** 1 Department of Medical Research Ministry of Health and Sports Pyin Oo Lwin Myanmar; 2 National Tuberculosis Programme Department of Public Health Ministry of Health and Sports Mandalay Myanmar; 3 Department of Epidemiology Faculty of Medicine Prince of Songkla University Hat Yai Thailand

**Keywords:** usability, mobile app, TB screening, chest X-ray compliance, mobile health, health application, risk score, tuberculosis, COVID-19

## Abstract

**Background:**

In Myanmar, the use of a mobile app for tuberculosis (TB) screening and its operational effect on seeking TB health care have not been evaluated yet.

**Objective:**

This study aims to report the usability of a simple mobile app to screen TB and comply with chest X-ray (CXR) examination of presumptive cases detected by the app.

**Methods:**

A new “TB-screen” app was developed from a Google Sheet based on a previously published algorithm. The app calculates a TB risk propensity score from an individual’s sociodemographic characteristics and TB clinical history and suggests whether the individual should undergo a CXR. The screening program was launched in urban slum areas soon after the COVID-19 outbreak subsided. A standard questionnaire was used to assess the app’s usability rated by presumptive cases. Compliance to undergo CXR was confirmed by scanning the referral quick response (QR) code via the app.

**Results:**

Raters were 453 presumptive cases detected by the app. The mean usability rating score was 4.1 out of 5. Compliance to undergo CXR examination was 71.1% (n=322). Active TB case detection among CXR compliances was 7.5% (n=24). One standard deviation (SD) increase in the app usability score was significantly associated with a 59% increase in the odds to comply with CXR (*β*=.464) after adjusting for other variables (*P*<.001).

**Conclusions:**

This simple mobile app got a high usability score rated by 453 users. The mobile app usability score successfully predicted compliance to undergo CXR examination. Eventually, 24 (7.5%) of 322 users who were suspected of having TB by the mobile app were detected as active TB cases by CXR. The system should be upscaled for a large trial.

## Introduction

In global practice, tuberculosis (TB) screening based on signs and symptoms is the first step in a case-finding strategy. Those having positive results should proceed to have their diagnosis confirmed with a chest X-ray (CXR) and sputum examination [1]. Previous analysis of TB screening data revealed that a TB risk propensity score calculated from sociodemographic and TB clinical variables could produce a better prediction of active TB than that based on TB signs and symptoms alone (80.6% vs 59.8% in sensitivity, 63.5% vs 67.2% in specificity, and 80.5% vs 63.7% in the area under the curve of a receiver operating characteristic curve) [2].

Based on these findings, a simple mobile health (mHealth) app was created and applied in an area close to where the score was developed. The app uses the statistics from Htet et al [2] to identify high-risk people who have a ≥0.5% probability to develop TB. They were then suggested to undergo CXR examination free of charge.

Standard mHealth app usability questionnaires have been developed and well tested [3]. For continuous improvement of the screening program, usability of the app needs to be assessed. In addition, to assess the operational effect of the app, it is important to follow up on the compliance of presumptive TB cases detected by the app and whether they would proceed to TB health center services for CXR examination as early as possible. In Myanmar, the use of a mobile app for TB screening and its effect on TB health services have not been assessed yet.

The aims of this study were (1) to assess the usability of our mHealth app perceived by presumptive TB cases, (2) to assess their compliance to proceed to undergo CXR examination, and (3) to determine the association between the usability of the app and compliance to undergo CXR examination.

## Methods

### Study Setting

This study was carried out in a low-income urban area of Mandalay City, which is densely populated and located in the central part of Myanmar [4-6]. Mandalay City has 7 township health departments providing TB services. Free medical services for TB diagnosis, including CXR and treatment, are available at TB health centers of the township health departments [7]. Routine TB care services are provided with precautions of COVID-19 prevention and control measures. For this study, 1 urban slum area was randomly selected from the list from each of 3 randomly selected townships by township medical officers and the regional TB coordinator.

### Developing a Mobile Health App

The “TB-screen” app was developed by AppSheet's no-code app (Seattle, WA, USA) [8]. It was a collaborative effort between the Department of Medical Research, National TB Control Programme, Myanmar, and the Department of Epidemiology, Prince of Songkla University, Hat Yai, Thailand.

The app was built on the principal investigator’s computer directly from Google Sheet. It was designed to be used off-line on a mobile device running an Android operating system (Google, Inc., Mountain View, CA, USA). The app was installed in the mobile phones of health care providers who are responsible for TB screening. They were carefully trained on how to use the mHealth app. They invited a family household with their neighbors to join TB screening. After entering sociodemographic and TB clinical variables of an individual via the app, the TB risk propensity score is computed using the following formula [9]:

TB risk propensity score = exp(BX)/[1 + exp(BX)],

where B is a vector of regression coefficients and X is the matrix of sociodemographic and TB clinical covariates of the subject. Details of the variables and their related coefficients are shown in Multimedia Appendix 1. It was noted that the coefficient of the elderly age groups and male groups were higher than all other variables. The oldest age group (55+ years) had an e^2.4^=11.0 times higher risk of developing TB than younger ones. The male group had an e^1.0^=2.07 times higher risk of developing TB than the female group. These groups had high potential to be identified as presumptive TB cases via the mobile app. The TB risk propensity score is the value that measures the risk of developing TB [2].

**Figure 1:**
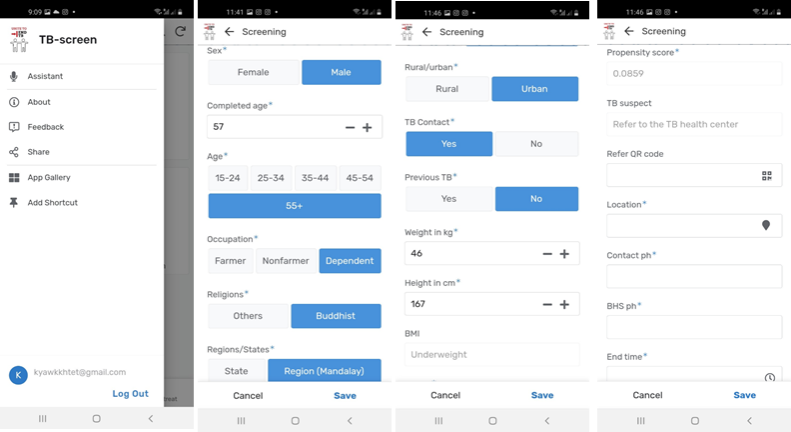
Screenshot of the TB-screen app. TB: tuberculosis.

With a selected propensity score cut-off level at ≥0.0052 (≥0.5% probability to develop TB), the test was taken as positive. The sensitivity is 80.6%, and the specificity is 63.5% [2]. A person with a positive test result, the presumptive TB case, is recommended to visit a TB health center for CXR examination. If they agree to go to the CXR center, a specific quick response (QR) code with a CXR referral form is scanned to the mHealth app (Figure 1). The presumptive TB case is given the CXR referral form to take to the CRX center. When the app is connected to the internet, the information collected is uploaded to the main server of data storage, with a notification to the corresponding TB health center. The data are updated again by the CXR center by scanning the referral QR code when the person visits and undergoes CXR examination. Figure 1 shows a screenshot of the TB-screen app.

### Ethical Considerations

The study was approved by the Institutional Ethics Committee of the Faculty of Medicine, Prince of Songkla University, Hat Yai, Thailand (REC:63-074-18-1), and the Institutional Review Board of the Department of Medical Research, Myanmar (IRB00008835).

### Screening Procedures

The screening process was carried out during November 2020-January 2021. This was after the COVID-19 outbreak substantially subsided and lockdowns and travel restrictions were eased. A trained local health care provider who is responsible for providing both TB and COVID-19 care in the assigned community performed TB screening via the app, following COVID-19 prevention and control measures. For each community, a family household with neighbors was approached for TB screening. Only 4 or 5 family households were approached, and at most 5-6 presumptive TB cases were referred for CXR examination per day to avoid overloading at the CXR center. The health care providers had been vaccinated with the complete dose of the COVID-19 vaccine. The presumptive TB cases were provided with face masks and face shields to be used during the interview and when they went to the CXR center. The presumptive TB cases with suspected COVID-19 were referred to the community fever clinic center at the respective township health department for COVID-19 investigation. Those who tested negative for COVID-19 proceeded to the CXR center for CXR examination.

At the CXR center, there was a separate waiting room for the presumptive TB cases from the same community, with the chairs arranged with social distancing measures. One CXR technician was assigned for the CXR examination during the project. After CXR examination was performed, the presumptive TB cases returned home without waiting for CXR results. They were informed about the CXR results by the health care provider who performed TB screening. The presumptive TB cases needed a single visit for CXR examination, and their remaining diagnostic and treatment procedures were aided by the health care provider and local health volunteers. The presumptive TB cases with abnormal suggestion of TB on CXR were confirmed as active TB cases by performing Gene Xpert *Mycobacterium tuberculosis* complex and resistance to rifampin (MTB/RIF) examination.

### Data Collection

During screening, informed consent for those >18 years old or guardian’s assent for those 15-18 years old was obtained for each participant. The screened participants were informed about survey processes using the app, and they were interviewed about their background, sociodemographic, and TB clinical characteristics; perceived susceptibility to developing TB; perceived benefits of TB screening; and perceived harms of TB screening. After collecting the variables related to the TB risk propensity score via the app, information about usability of the mHealth app was collected. Point coordinate data for location of participants were taken by the app for further use to calculate the distance between their residences and the TB health center for CXR. Accessibility methods to the CXR center were also recorded.

### Statistical Analysis

#### Proposed Model of the Analysis

**Figure 2:**
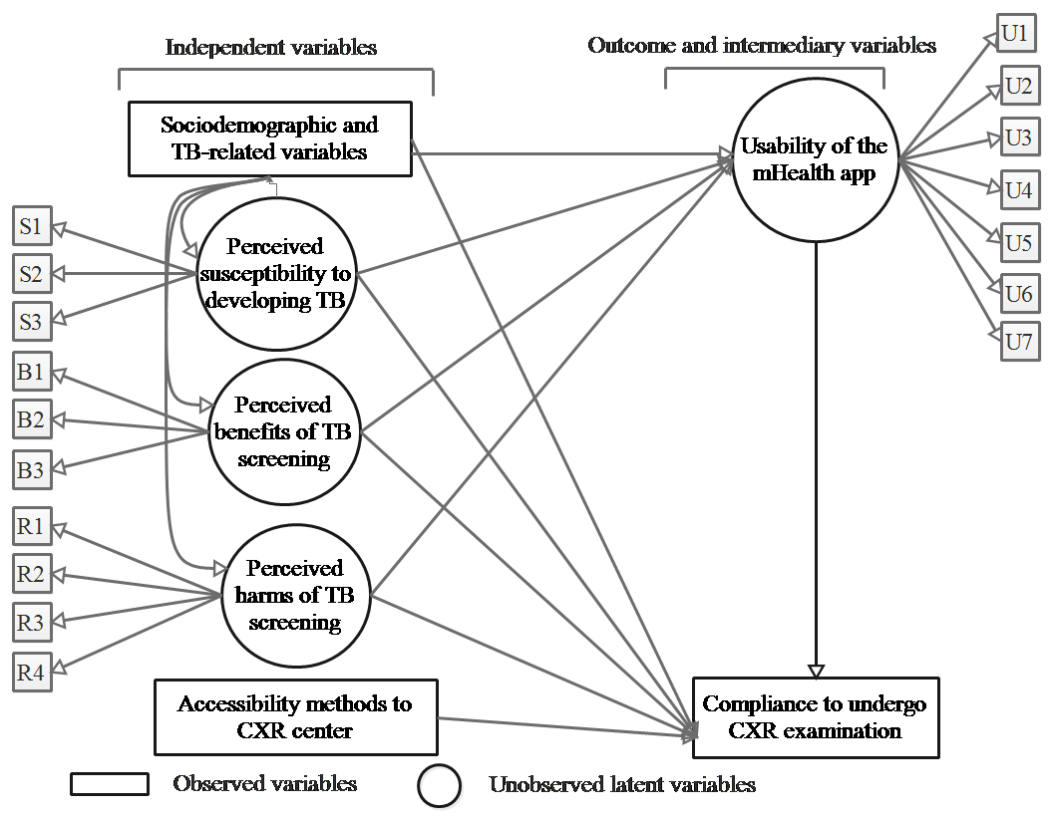
Baseline model to determine the association between usability of the app and compliance to undergo CXR examination and their influencing factors. Small boxes with U, S, B, and R denote items measured for the respective latent variable. CXR: chest X-ray; TB: tuberculosis.

Figure 2 illustrates the proposed major relationships among the variables and their components. The usability of the app was an intermediary latent variable influenced by background, sociodemographic, and clinical variables, which also influenced 3 latent variables, namely perceived susceptibility to developing TB, perceived benefits of TB screening, and perceived harms of TB screening. All these variables, including usability of the app, influenced compliance to CXR examination. Finally, compliance to CXR examination was also influenced by accessibility methods to a CXR center.

#### Outcome Variable

The primary outcome was compliance of presumptive TB cases detected by the app to undergo CXR examination within 1-7 days of TB screening.

#### Intermediary Variable

The main intermediary variable was the usability of the mHealth app, which was a latent variable. It was measured by using the standard mHealth app usability questionnaire (U1-U7 items, Cronbach *α*=.9) [3].

#### Independent Variables

The sociodemographic and TB-related variables included marital status, education, family income per month (US $), the TB risk propensity score, TB signs and symptoms, and knowledge of TB.

The TB risk propensity score was derived from age, gender, occupation, religion, area of residence, administrative division, contact with a known TB case, previous history of TB, and BMI (kg/m^2^) [2].

TB signs and symptoms included cough, hemoptysis, recent loss of weight, chest pain, and fever within the previous 1 month. There were 5 TB knowledge questions adopted from the World Health Organization (WHO) TB survey, which asked about TB signs and symptoms, persons who are at high risk of developing TB, transmission, diagnosis methods, and cure [10]. A correct answer was assigned a score of 1, and 0 otherwise.

Items related to perceived susceptibility to developing TB, perceived benefits of TB screening, and perceived harms of TB screening via the app were constructed using Champion and Skinner’s variable definitions [11]. The perceived susceptibility to developing TB was assessed by 3 items (S1-S3); for example, “You are at high risk of TB infection.” The perceived benefits of TB screening were constructed by 3 items (B1-B3); for example, “TB screening is good for your health.” The perceived harms of TB screening were identified by 4 items (R1-R4); for example, “You are afraid of developing TB.”

The variables of accessibility methods to accessing a CXR center were availability to go to the center during clinic opening hours, having one’s own vehicle to go to the CXR center, and traveling distance (km) to the CXR center.

#### Confirmatory Factor Analysis

Confirmatory factor analysis (CFA) was performed to verify the fit of the observed items, internal consistency, and discriminant validity to each latent variable [12-15].

#### Structural Analysis of the Causal Pathway in Structural Equation Modeling

The proposed model was analyzed by structural equation modeling (SEM). Based on the construction illustrated in Figure 2, the structural analysis of SEM was used to examine whether our baseline proposed model was acceptable and a good fit [16]. The independent variables having a *P* value ≥.05 were dropped each time until the goodness-of-fit was acceptable. The model is acceptable when the Tucker-Lewis fit index (TLI)≥0.95, the ratio of the chi-square statistic to its degrees of freedom is close to 1, cumulative fit index (CFI)≥0.95, root-mean-square error of approximation (RMSEA)≤0.05, and standardized root-mean-square residual (SRMR)≤0.08 [12-14].

In the final fitted SEM model, the standardized estimate (*β*), regression coefficient, and standard error (SE) of observed variables and latent variables associated with usability of the app and compliance to undergo CXR examination were calculated. The data were analyzed using R version 4.0.0 (R Foundation for Statistical Computing) [17].

## Results

### Subject Classification

**Figure 3:**
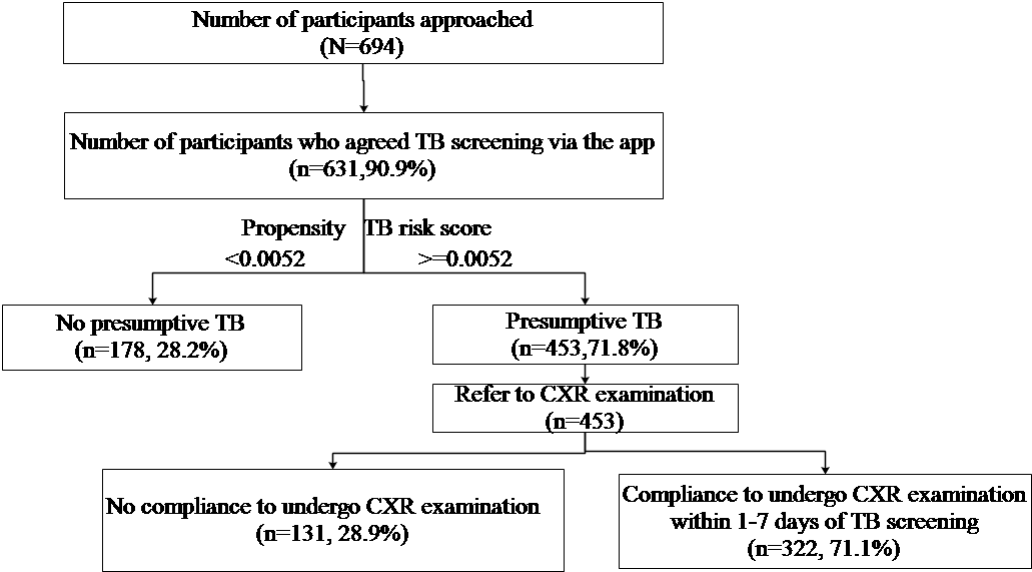
Flowchart of participants under TB screening until visit for CXR examination. CXR: chest X-ray; TB: tuberculosis.

Figure 3 summarizes the overall results of subject classification based on the TB risk propensity score among the screened participants and compliance to undergo CXR examination among the presumptive TB cases. Of 694 participants approached, 631 (90.9%) agreed to be screened via the app. Among those screened, 453 (71.8%) were identified as presumptive TB cases by the app and were suggested to visit a TB health center for a CXR appointment.

### Compliance to Undergo CXR Examination

As shown in Figure 3, of 453 presumptive TB cases, 322 (71.1%) complied to undergo CXR examination within 1-7 days of TB screening.

### Characteristics of Presumptive TB Cases Detected by the App (N=453)

Table 1 shows the background characteristics of the 453 presumptive TB cases. Males and the elderly had a high proportion, accounting for 251 (55.4%) and 150 (33.1%) participants, respectively. The mean (SD) age was 46.1 (15.0) years. The median TB risk propensity score was 0.01 (IQR 0.0058-0.022). A high risk score indicated an increase in the risk of developing TB. Over half (n=258, 57.0%) had no TB signs or symptoms. Knowledge of TB was high, with a mean (SD) score of 5.9 (1.4) out of 8. The cases had less perceived susceptibility to developing TB, with a mean (SD) score of 2.7 (1.0) out of 5, but more perceived harms of TB screening, with a mean (SD) score of 3.0 (1.1) out of 5.

**Table 1:** Characteristics of the presumptive TB^a^ cases (N=453).

Variables and their description		Value
**Marital status, n (%)**		
	Married	403 (89.0)
	Single	50 (11.0)
**Education, n (%)**		
	None	25 (5.5)
	Primary school	220 (48.6)
	Secondary school	101 (22.3)
	Middle school	71(15.7)
	High school and above	36 (7.9)
**Family income per month (US $), n (%)**		
	≤80	112 (24.7)
	81-240	320 (70.6)
	241-400	17 (3.8)
	>400	4 (0.9)
**Gender, n (%)**		
	Female	202 (44.6)
	Male	251 (55.4)
**Age (years), n (%)**		
	15-24	29 (6.4)
	25-34	82 (18.1)
	35-44	106 (23.4)
	45-54	86 (19.0)
	+55	150 (33.1)
Age (years), mean (SD)		46.1 (15.0)
**Religion, n (%)**		
	Buddhist	438 (96.7)
	Others	15 (3.3)
**Occupation, n (%)**		
	Dependent	218 (48.1)
	Farmer	21 (4.7)
	Nonfarmer	214 (47.2)
**Contact with a known TB case, n (%)**		
	No	291 (64.2)
	Yes	162 (35.8)
**Previous history of TB, n (%)**		
	No	353 (77.9)
	Yes	100 (22.1)
**TB signs and symptoms, n (%)**		
	Yes	195 (43.0)
	No	258 (57.0)
BMI (kg/m^2^), mean (SD)		19.8 (3.3)
TB risk propensity score, median (IQR)		0.01 (0.0058-0.022)
Knowledge of TB (range 0-8), mean (SD)		5.9 (1.4)
Perceived susceptibility to developing TB^b^, mean (SD)		2.7 (1.0)
Perceived benefits of TB screening^b^, mean (SD)		4.4 (0.6)
Perceived harms of TB screening^b^, mean (SD)		3.0 (1.1)
**Available to go to CXR^c^ center during clinic opening hours, n (%)**		
	Yes	289 (63.8)
	No	164 (36.2)
**Have an own vehicle to go to the CXR center, n (%)**		
	Yes	93 (20.5)
	No	360 (79.5)
**Distance to CXR center (km), n (%)**		
	≤10	211 (46.6)
	>10	242 (53.4)

^a^TB: tuberculosis.

^b^Latent variables.

^c^CXR: chest X-ray.

### Usability of the Mobile Health App

Table 2 shows the usability scores of the mHealth app rated by the presumptive TB cases. All items had an average rating score of >3. The overall rating on the usability of the mHealth app was favorable, with a mean (SD) score of 4.1 (1.1) out of 5, indicating a high level of usability rated by the users.

**Table 2:** Usability of the mHealth^a^ app by the presumptive TB^b^ detected by the app (N=453).

Item	Strongly disagree	Disagree	Neutral	Agree	Strongly agree	Mean (SD)	Cronbach *α*=.941
	1	2	3	4	5		
U1: The mobile app improves your access to TB health care services.	0	61	31	181	180	4.0 (1.0)	N/A^c^
U2: The mobile app makes it convenient for you to communicate with your health care provider.	0	79	14	145	215	4.1 (1.1)	N/A
U3: By using the mobile app in TB screening, you have many more opportunities to interact with the health care provider.	0	80	13	130	230	4.2 (1.1)	N/A
U4: You feel confident that any information you received from the mobile app.	0	51	44	145	213	4.0 (1.0)	N/A

^a^mHealth: mobile health.

^b^TB: tuberculosis.

^c^N/A: not applicable.

### Confirmatory Factor Analysis

In Table 3, the final CFA showed a good fit after removing 3 items from the usability of the app (U5, U6, and U7) and 2 items from the perceived harms of TB screening (R3 and R4) with factor loadings of <0.3 in the baseline CFA. Each latent variable was rated using a 5-point Likert scale ranging from 1 for “totally disagree” to 5 for “totally agree.”

Table 4 shows that the composite reliability was higher than the average variance extracted and the average variance extracted was >0.5. In addition, the square root of the average variance extracted was higher than the correlations of the latent variables under analysis. Thus, the latent variables had both good consistency and discriminant validity.

**Table 3:** CFA^a^ of latent variables.

Items		Baseline			Final		
		Factor loading	Cronbach *α*	Model fit (*χ*^2^_(113)_=1078.3, *P*<.001, CFI^b^=0.797, TLI^c^=0.756, RMSEA^d^=0.137, SRMR^e^=0.099)	Factor loading	Cronbach *α*	Model fit (*χ*^2^_(47)_=74.1, *P*=.01, CFI=0.993, TLI=0.99, RMSEA=0.036, SRMR=0.026)
**Usability of the mHealth^f^ app**		N/A^g^	.7919	N/A	N/A	.941	N/A
	U1: The mobile app improves your access to TB^h^ health care services.	0.872	N/A	N/A	0.872	N/A	N/A
	U2: The mobile app makes it convenient for you to communicate with your health care provider.	0.764	N/A	N/A	0.764	N/A	N/A
	U3: By using the mobile app in TB screening, you have many more opportunities to interact with the health care provider.	0.953	N/A	N/A	0.953	N/A	N/A
	U4: You feel confident that any information you received from the mobile app.	0.961	N/A	N/A	0.961	N/A	N/A
	U5: The app is useful for improving your health and well-being.	0.096	N/A	N/A	N/A	N/A	N/A
	U6: You feel comfortable communicating with your health care provider using the app.	0.018	N/A	N/A	N/A	N/A	N/A
	U7: The app helps you manage your health effectively.	0.058	N/A	N/A	N/A	N/A	N/A
**Perceived susceptibility to developing TB**		N/A	.921	N/A	N/A	.921	N/A
	S1: You are at high risk of TB infection.	0.936	N/A	N/A	0.936	N/A	N/A
	S2: You are probably infected with TB with or without having TB signs or symptoms.	0.850	N/A	N/A	0.850	N/A	N/A
	S3: You are the most possible person to be infected with TB among all family members.	0.894	N/A	N/A	0.894	N/A	N/A
**Perceived benefits of TB screening**		N/A	.730	N/A	N/A	.730	N/A
	B1: This screening tool is convenient to identify TB early.	0.712	N/A	N/A	0.712	N/A	N/A
	B2: If results of the screening are positive, you can access a TB health center for early TB diagnosis.	0.877	N/A	N/A	0.877	N/A	N/A
	B3: TB screening is good for your health.	0.502	N/A	N/A	0.502	N/A	N/A
**Perceived harms of TB screening**		N/A	.608	N/A	N/A	.926	N/A
	R1: You are afraid of developing TB.	0.953	N/A	N/A	0.978	N/A	N/A
	R2: You are afraid of suffering social stigma due to TB.	0.905	N/A	N/A	0.882	N/A	N/A
	R3: Screening by using the mobile app protects your privacy.	0.159	N/A	N/A	N/A	N/A	N/A
	R4: Screening by using the mobile app keeps your personal information confidential.	–0.033	N/A	N/A	N/A	N/A	N/A

^a^CFA: confirmatory factor analysis.

^b^CFI: comparative fit index.

^c^TLI: Tucker-Lewis index.

^d^RMSEA: root-mean-square error of approximation.

^e^SRMR: standardized root-mean-square residual.

^f^mHealth: mobile health.

^g^N/A: not applicable.

^h^TB: tuberculosis.

**Table 4:** Internal consistency and discriminant validity of latent variables in the fitted CFA^a^.

Latent variables	Items	Composite reliability	Average variance extracted	Correlation coefficients			
				Usability of the mHealth^b^ app	Perceived susceptibility to developing TB^c^	Perceived benefits of TB screening	Perceived harms of TB screening
Usability of the mHealth app	4	0.896	0.764	1	N/A^d^	N/A	N/A
Perceived susceptibility to developing TB	3	0.922	0.797	(–0.042)	1	N/A	N/A
Perceived benefits of TB screening	3	0.754	0.521	(0.058)	(0.001)	1	N/A
Perceived harms of TB screening	2	0.929	0.867	(–0.24)	(–0.088)	(0.01)	1

^a^CFA: confirmatory factor analysis.

^b^mHealth: mobile health.

^c^TB: tuberculosis.

^d^N/A: not applicable.

### Structural Analysis of the Causal Pathway in SEM

As shown in Table 5, the final SEM model was acceptable (TLI>0.95) and showed a better fit than the baseline proposed model (CFI=0.955 vs 0.922).

**Figure 4:**
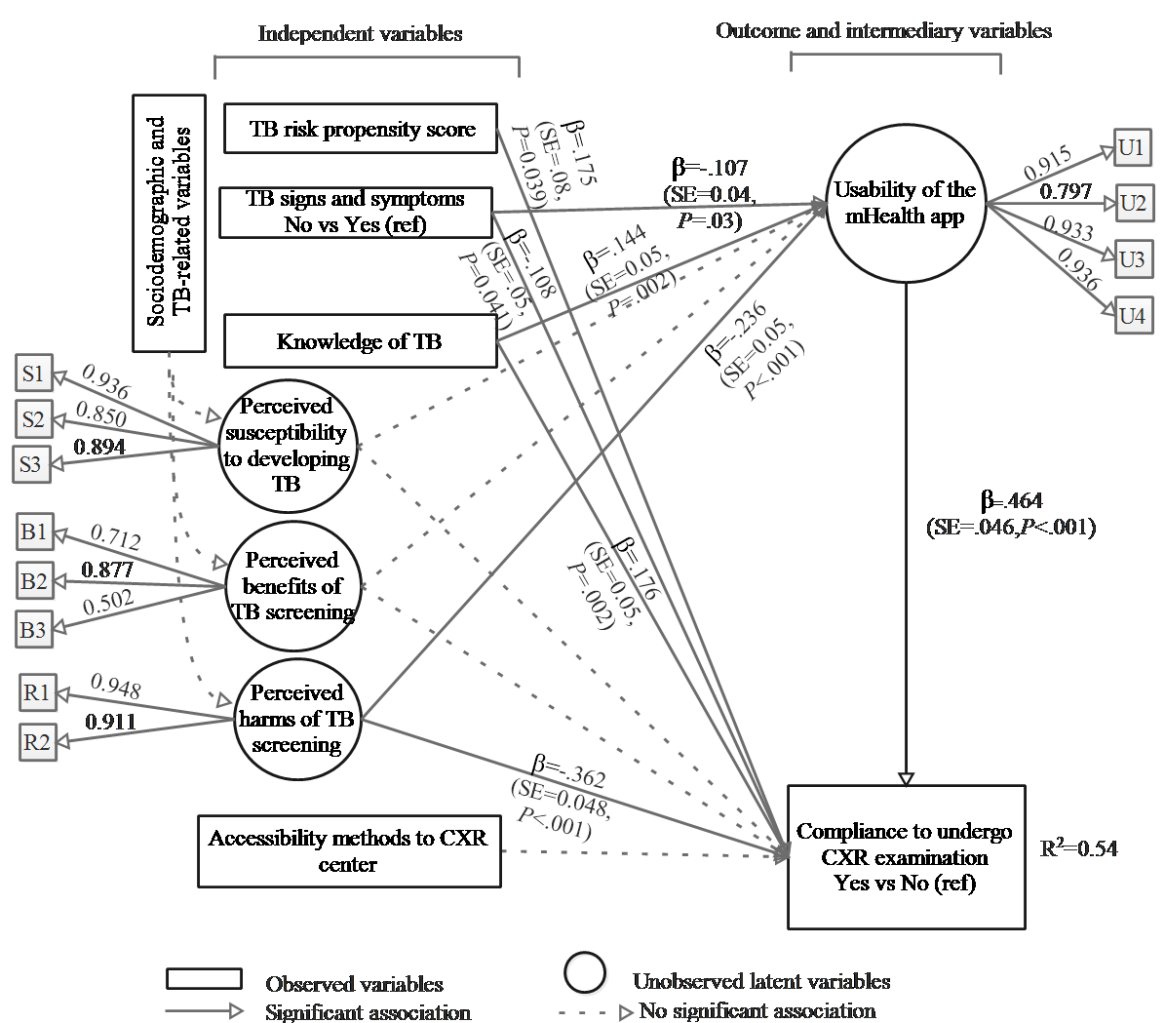
Standardized coefficients (*β*) of variables in structural analysis of the causal pathway in the final fitted SEM. Note: Nonsignificant variables from sociodemographic variables, TB-related variables, and accessibility methods to CXR centers were dropped from the final SEM. Small boxes with U, S, B, and R denote items measured for the respective latent variable. CXR: chest X-ray; SEM: structural equation modeling; TB: tuberculosis.

Figure 4 shows the standardized coefficients (*β*) of variables in the structural analysis of the causal pathway in the final SEM.

In the final SEM, the usability of the mHealth app and compliance to undergo CXR examination were not associated with marital status, education, perceived susceptibility to developing TB, perceived benefits of TB screening, and variables of accessibility methods to the CXR center. For clarity, these variables are not shown in Figure 4.

The usability of the mHealth app was significantly associated with compliance to undergo CXR examination. As *β*=.464, 1 standard deviation increase in the usability score of the app would be associated with e^0.464^=1.59-fold odds or 59% increase to comply to undergo CXR examination.

The other significant factors associated with compliance were a high TB risk propensity score and high TB knowledge. In contrast, those with a lack of TB signs and symptoms and with perceived harms of TB screening were less likely to comply to undergo CXR examination.

Similarly, having high TB knowledge favored the usability of the app, while a lack of TB signs and symptoms and having perceived harms of TB screening predisposed individuals to be less favorable toward the usability of the app.

**Table 5:** Comparison of baseline and final SEM^a^ models.

SEM comparison	*χ*^2^ (*df*)	*P* value	CFI^b^	TLI^c^	RMSEA^d^ (90% CI)	SRMR^e^
Baseline	255.6 (143)	<.001	0.922	0.957	0.042 (0.033-0.05)	0.04
Final	52.9 (34)	.02	0.955	0.972	0.035 (0.014-0.053)	0.025

^a^SEM: structural equation modeling.

^b^CFI: comparative fit index.

^c^TLI: Tucker-Lewis index.

^d^RMSEA: root-mean-square error of approximation.

^e^SRMR: standardized root-mean-square residual.

### Active TB Case Detection Among Presumptive TB Cases Who Complied to Undergo CXR Examination

Of 453 presumptive TB cases detected by the app, 322 (71.1%) complied to undergo CXR examination. Of them, 56 (17.4%) were identified as showing an abnormal suggestion of TB in the CXR and continued for Gene Xpert MTB/RIF examination. The total active TB case detection was 24 (7.5%) of 322 presumptive TB cases.

## Discussion

### Principal Findings

This is the first study in Myanmar using a mobile app for screening for TB and assessing its impact on seeking TB health care services. This mHealth app was rated by community members to have a good usability score. A significant proportion of the presumptive TB cases were identified via the app. Nearly three-quarters of the presumptive TB cases identified by the app proceeded to undergo CXR examination, and 7.5% showing CXR compliance were diagnosed as active TB cases. The usability of the mHealth app was the strongest predictor associated with the compliance to undergo CXR examination. Low predicting variables included TB signs and symptoms, perceived harms of TB screening, the TB risk propensity score, and knowledge of TB.

In this study, a high number of presumptive TB cases were identified via the mHealth app and TB health centers were notified. This could be explained by the high percentages (55.4% and 33.1%) of subjects under screening being male and elderly, respectively. The setting of the app gave a high score to these groups. In addition, nearly three-quarters of the presumptive TB cases identified by the app complied to undergo CXR examination. It was relatively high compared to a previous study in Myanmar [18]. Eventually, 24 (7.5%) of 322 users who were suspected by the app as having TB were detected as active TB cases by CXR. This proportion was missing active TB cases in the community that were early detected by the mobile app. However, this achievement would not be possible without the compliance to undergo CXR examination by the presumptive TB cases detected by the app. Completion of this step would lead to early detection of TB cases, achieving the WHO-recommended “universal access to test and treat process” [19].

During implementation of the app, the participants rated the usability of the mHealth app highly and a significant proportion of presumptive TB cases were detected via the app. Many studies have revealed patients’ acceptance of using mHealth apps in TB care to improve health outcomes [20,21]. In addition, the favorable rating for the usability of the mHealth app had a positive association with compliance to undergo CXR examination. In this study, the mobile app for TB screening was integrated into a pathway to TB diagnosis by notifying the CXR center of screened positive cases. This integration might be the reason for the high association of these two variables. A similar improvement in TB referrals by engaging the presumptive TB cases in the health sector via the app was shown in a study in India [22].

In this study, presumptive TB cases without TB signs or symptoms and those who had perceived harms of TB screening gave a less favorable rating to the usability of the app and had poor compliance to undergo CXR examination. A review of national TB prevalence surveys in Asia highlighted that those who screened negative for TB symptoms were less likely to seek TB care until symptoms worsened [23]. Many studies have revealed that having perceived harms of TB screening has a negative impact on TB screening and subsequent diagnostic procedures [24-26]. Having knowledge of TB can increase acceptance of patients with TB and encourage subsequent screening and diagnostic processes [27].

### Strengths and Limitations

Compared to similar studies, this mHealth app calculated the TB risk propensity score instead of using TB signs and symptoms [22,28-30]. Although the TB risk propensity score has better TB prediction than TB signs and symptoms, there is a limitation in applying it in the real field, probably because of its complexity to calculate [31,32]. However, our mobile app overcomes this gap.

In addition, a review of a national TB prevalence survey in Asia (1990-2012) revealed that 40%-60% of active TB cases are missed by screening for routine TB signs and symptoms, because this proportion of patients are asymptomatic and not identified as presumptive TB cases [23]. In Myanmar, the TB case detection rate by screening for routine TB signs and symptoms was 69%-77% in 2017-2019 [33-35]. This highlighted that nearly one-fourth of active TB cases were missing, either not notified to the national TB program or not diagnosed and treated. Therefore, using a mobile app that calculates the TB risk score is a potential new approach to identifying missing presumptive TB and active TB cases that would not be detected by TB signs and symptoms only.

As strengths, the mobile app was used to calculate the TB risk propensity score so misclassification bias to identify the TB suspects was reduced. Real-time data entry was performed for the CXR examination date so that data error was less likely in assessing compliance. The app is simple to develop, provided that existing TB survey databases can be used to calculate the TB risk propensity score for the population under study. This idea can be adapted to many low-resource countries.

However, a high level of compliance to undergo CXR examination in this study was probably due to worry about long COVID among the population after a serious COVID-19 outbreak recently subsided [36]. In addition to TB detection, they might expect CXR to detect the residual effects of COVID-19 and thus they had high compliance. As a limitation, high compliance to undergo CXR examination may suggest that the TB screening program could be done better immediately after a COVID-19 outbreak or its generalizability should be confirmed when the COVID-19 situation is no more perceived as a serious threat.

### Conclusion

The simple mobile app we developed got a high usability score by 453 users. The mobile app usability score successfully predicted compliance to undergo CXR examination. Eventually, 24 of 322 users who were suspected by the mobile app as having TB were detected as active TB cases by CXR. The system should be upscaled for a large trial.

## Appendix

Multimedia Appendix 1 Regression coefficient (*β*) of significant sociodemographic and TB clinical history variables to predict bacteriologically confirmed TB. TB: tuberculosis.

## Abbreviations

CFA: confirmatory factor analysis
CFI: cumulative fit index
CXR: chest X-ray
mHealth: mobile health
QR: quick response
RMSEA: root-mean-square error of approximation
SEM: structural equation modeling
SRMR: standardized root-mean-square residual
TB: tuberculosis
WHO: World Health Organization
