# Supplementary material for: Mobile Health App for Tuberculosis Screening and Compliance to Undergo Chest X-ray Examination Among Presumptive Cases Detected by the App in Myanmar: Usability Study
Source: JMIR Form Res. 2022 Jun 7;6(6):e37779. doi: 10.2196/37779 (PMC9177170; doi:10.2196/37779)
Supplement: Multimedia Appendix 1 [file formative_v6i6e37779_app1.docx]

**Appendix 1**

Regression coefficients (β) of significant socio demographic and TB clinical variables to predict bacteriologically confirmed TB

| Variables that predict TB | Description | Coefficient (β) |
| --- | --- | --- |
| Intercept |  |  |
|  |  | -8.4746 |
| Age group (years) |  |  |
|  | 15-24 | 0.0000 |
|  | 25-34 | 1.8248 |
|  | 35-44 | 2.3756 |
|  | 45-54 | 2.3916 |
|  | 55+ | 2.4088 |
| Gender |  |  |
|  | Female | 0.0000 |
|  | Male | 1.0436 |
| Occupation |  |  |
|  | Non farmer | 0.0000 |
|  | Farmer | -0.2130 |
|  | Dependent | 0.4323 |
| Religion |  |  |
|  | Buddhist | 0.0000 |
|  | Other | 0.4893 |
| Area of residence |  |  |
|  | Rural | 0.0000 |
|  | Urban | 0.4520 |
| Administrative division |  |  |
|  | Region | 0.0000 |
|  | State | 0.4664 |
| Contact with a known TB case |  |  |
|  | No | 0.0000 |
|  | Yes | 0.7214 |
| Previous history of TB |  |  |
|  | No | 0.0000 |
|  | Yes | 1.0313 |
| Body mass index |  |  |
|  | Underweight | 1.0519 |
|  | Normal | 0.0000 |
|  | Overweight/obese | -0.9069 |

^TB=Tuberculosis^
